# The Role of Multi Detector Computed Tomographic Imaging Prior to Clinic Consultation in Patients Suspected to Have Lung Cancer

**DOI:** 10.4021/wjon513w

**Published:** 2012-07-05

**Authors:** Vimal Raj, Sumit Gupta, Tamilselvan Sivashanmugam, Daniel Barnes, Sanjay Agrawal, Jonathan A Bennett, Michael D Peake, James J Entwisle

**Affiliations:** a Department of Radiology, Glenfield Hospital, University Hospitals of Leicester, Groby road, Leicester, LE3 9QP, UK; b Department of Medicine, Glenfield Hospital, University Hospitals of Leicester, Groby road, Leicester, LE3 9QP, UK

**Keywords:** Lung cancer, MDCT, Screening

## Abstract

**Background:**

Scanning patients early in their diagnostic journey helps in differentiating benign from malignant aetiology. There is increasing pressure on diagnostic practices for rapid diagnoses and thereby early commencement of treatment in patients suspected to have lung cancer (LC). In our practice, multi detector computed tomography (MDCT) imaging is performed in selected patients referred to the LC service prior to them seeing a chest physician in the LC clinic. This study evaluates the role of such practice and reviews its potential impact on LC services.

**Methods:**

Prospective review of our practice from January 2007 to Apr 2007 was performed. Consecutive patients referred to the service with suspected LC were included. Chest radiograph (CXR) report and clinical information from general practitioners were reviewed and graded as high, medium or low risk for presence of LC. Patients with sufficient clinical and/or radiological concern underwent MDCT imaging prior to their clinic. Combined risk scores and modified risk scores were formulated and assessed against MDCT findings.

**Results:**

A total of 139 patients were referred to the service, 124 of these had pre-clinic MDCT. Fifty-three patients (43%) had malignancy, 39 (31%) had non-malignant significant abnormalities, 17 (14%) had other incidental findings and 15 (12%) were normal. Modified combined risk score was the best predictor of presence of cancer.

**Conclusion:**

Pre-clinic MDCT scanning in patients with suspected LC is feasible and has a promising role in the modern care of LC patients. It also empowers physicians with additional information at the primary consultation.

## Introduction

Lung Cancer (LC) is one of the leading causes of mortality and morbidity in United Kingdom. Early detection of disease is associated with better prognosis. Multiple LC screening programmes are active in Europe and USA aimed at early detection of the disease in high-risk patient population [1-6]. Early follow up data coming out of these studies however; show that the effect on mortality might be smaller than expected [3]. It may be decades before LC screening is widely offered in NHS, until then it is vital that all possible steps are taken to ensure early detection of LC and prompt initiation of treatment. ‘The NHS Cancer plan’ was conceived in 2000, with an aim to reduce death rates and improve prospects of survival by improving prevention, promoting early detection and effective screening practice [7]. This led to the establishment of multiple targets aimed at achieving these goals. Therefore, recognition and avoidance of any source of delay between presentation and treatment remains a primary objective not only for the patient but also for the hospital trusts.

Patients diagnostic journey starts with their visit to the General Practitioner (GP). Those who have symptoms and/or signs suspicious of underlying LC will have a chest radiograph (CXR) initially. If CXR is abnormal and/or if there are concerning symptoms, the patient is urgently referred to the respiratory/LC clinic by the GP. In many centres, patient is then seen in the out patient clinic and invariably referred for further investigations such as multi detector computed tomography (MDCT) of the chest/abdomen, ultrasound, bronchoscopy etc. It has been shown that scanning the patient early in their diagnostic journey helps to differentiate benign from malignant diseases and can direct further diagnostic investigations [8].

Therefore, in our centre we follow a specific pathway for patients referred to the rapid access lung (RAL) clinic. Carefully selected patients, based on CXR report and/or clinical presentation, undergo MDCT imaging prior to their initial assessment by the respiratory physicians at the RAL clinic.

## Methods

### Objective

To evaluate the role of performing MDCT imaging in patients suspected to have LC prior to their assessment by respiratory physician at the RAL clinic.

Produce an algorithm that will guide clinicians in requesting appropriate scan for an individual patient in this clinical setting.

### Setting

In our centre all CXR’s suspicious of LC are brought to the urgent attention of RAL clinic coordinator and the referring clinician, usually GP. The patient is then referred to the RAL clinic by the GP or responsible clinician. The respiratory physicians review the clinical details and CXR report and the patient is triaged into two categories: 1) Category A: Review in RAL clinic (patient is seen in the clinic by the physicians); 2) Category B: MDCT imaging prior to clinic consultation.

### Study design

Data was collected prospectively between January and April 2007. All patients referred to the RAL clinic with suspected LC were included. Demographic data, clinical symptoms and CXR findings were collated. To produce an algorithm tailored to individual patient, all patients had their CXR report and clinical information subjectively graded (prior to staging scan) as high, medium or low risk for suspected LC by a respiratory physician. From these clinical risk score (CRS) and modified clinical risk scores (MCRS) were formulated.

### Scan technique

Scans were acquired on a MDCT scanner (Somatom Sensation 16, Siemens AG, Germany) with 1.5 mm collimation and a reconstruction interval of 2 mm. Images of the thorax were acquired at 25 seconds post intravenous contrast and the upper abdomen images were acquired at 60 seconds delay with sufficient overlap. Images were reconstructed on standard mediastinal and lung window algorithms.

### Statistical analysis

Statistical analysis was performed using GraphPad Prism (version 5.00 for Windows, GraphPad Software, USA) and SPSS (for Windows, Chicago: SPSS Inc). Parametric data were expressed as mean (SEM). Unpaired t test was used to compare the means of two unmatched groups. Chi squared tests were used to compare categorical data. Logistic regression analysis and reporting were performed as described previously [9]. Variables entered into the regression model were, age and MCRS. Contrast method used for MCRS, a categorical predictor variable in the regression model, was ‘repeated’ contrast method where each category of the predictor variable except the first category is compared to the category that precedes it. The final multivariate logistic regression model was determined, using block entry of variables, to assess factors, which best predicted presence of lung cancer. A p value of < 0.05 was taken as the threshold for statistical significance.

## Results

### Participants

Over the four-month period, 139 patients were referred to the RAL clinic. Average age was 67 years with more men (89) than women (50). Of these, 13 (9%) were reviewed in the clinic while 126 were referred for pre-clinic MDCT staging. Of the 126 patients referred for staging, 2 refused to have any imaging and 124 had MDCT. Of the 13 initially seen in the clinic, 7 went on to have a MDCT scan at a later date while 3 were followed by CXR and 3 managed without further imaging.

### Main results

The main results are outlined in Table 1. Fifty-three patients (43%) had a malignancy, 39 (31%) had other significant abnormality, 17 (14%) had insignificant abnormality and 14 scans (12%) were normal. There was no significant difference in the mean age of patients with and without malignancy. However with increasing age there was increase in detection of significant abnormality on MDCT scan (Chi-square, P = 0.016).

**Table 1:** Showing the Distribution of Patients Into Various Categories and the Respective Risk Scores

n = 124	Malignancy 53 (43%)		No Malignancy 71 (57%)	
Age (mean (SEM))*	69 (1.52)		67 (1.59)	
Abnormality in %	Lung	89	None	21
	Metastasis	7	Insignificant	24
	Others	4	Significant	55
CXR score in %	0	0	0	21
	1	23	1	24
	2	77	2	55
Clinical risk score in %	0	2	0	31
	1	36	1	63
	2	62	2	6
MCRS∧ in %	0-2 (Low)	25	0-2 (Low)	85
	3 (Intermediate)	13	3 (Intermediate)	10
	4 (High)	62	4 (High)	6

*unpaired t test, P = ns; ∧chi-square test, P < 0.0001.

Malignancies included primary lung cancer (47), metastatic lung disease (4) and one each of lymphoma and primary oesophageal malignancy. Significant abnormalities seen in patients with no malignancy included, 12 solitary pulmonary nodules (SPN), 16 cases of consolidation, 5 cases of sarcoid/unexplained adenopathy and six had tuberculosis, multiple pulmonary emboli, eosinophilic granuloma, cardiomyopathy, pulmonary hypertension and a vascular malformation. Other incidental findings included emphysema (7), atelectasis (3), benign rib lesions (2) and benign pleural disease in five patients.

### Analysis

The risk assessments for LC were stratified into numeric groups of 0 (low), 1 (medium) and 2 (high) for both CXR report and clinical risk scores. Combined risk score (CRS) was formulated by adding the above scores. ROC (Receiver Operating Characteristic) curve was used to assess the performance and accuracy of CRS in classifying patients into malignant and non-malignant group. The area under the curve was 0.820 (95% CI 0.745 to 0.894, P < 0.0001) and there was a good balance between sensitivity (75.5) and specificity (84.5) of the test with a CRS of > 2.

To make this simpler, CRS was modified (modified combined risk score (MCRS)) to stratify patients into three categories as: 1) CRS: 0-2 - Low risk (L); 2) CRS: 3 - Intermediate risk (I); 3) CRS: 4 - High risk (H).

Logistic regression analysis was performed with age and MCRS as variables. MCRS best predicted the presence of malignancy with significant decrease in the odds of detecting malignancy with change in MCRS form: M to L and H to M (Table 2).

**Table 2:** Logistic Regression Showing Significant Increase in Risk of Malignancy With Increasing MCRS and no Significant Influence of Age on Prediction of Malignancy

Logistic Regression Dichotomous dependent variable: Lung cancer				
Predictor variables	B (SE)	Wald’s χ^2^	P	Odds Ratio (95% CI)
Age	0.023(0.020)	1.371	0.242	1.024 (0.984 - 1.065)
MCRS		36.270	< 0.0001	
MCRS (1) (L with M)	-1.509 (0.621)	5.900	0.015	0.221 (0.065 - 0.747)
MCRS (2) (M with H)	-2.191 (0.764)	8.225	0.004	0.112 (0.025 - 0.500)

Goodness-of-fit test: Hosmer and Lemeshow; χ^2^ = 11.576, P = 0.171; Nagelkerke R^2^ = 0.499, c-statistic = 84.9%; Model accuracy in classification = 81.5%, Improvement in classification from baseline = 24.2%; Sensitivity = 69.81%, Specificity = 90.14%, PPV = 84.09%, NPV = 80%.

Based on above, an imaging algorithm was developed incorporating MCRS and the age of the patient (Table 3). In young patients with intermediate risk and older patients with low risk a ‘focused CT thorax’ (low dose without contrast) is recommended. A radiologist reviews these scans and if significant abnormality is detected, further appropriate imaging is performed at the same appointment. If this algorithm was put to use in our study group who had the staging scan, 69 (55%) patients would have had focused CT thorax, 4 (3%) reviewed in the clinic and 51 (41%) would have had staging (Chest and Abdomen) scan. None of the patients reviewed in the clinic would have had cancer, 78% of staging scans would be positive for malignancy and 10% patients would have needed staging scan after the focused CT thorax.

**Table 3:** Algorithm Incorporating MCRS and Age

Modified Combined risk score (MCRS)	Below 40 yrs	Above 40 yrs
High (4)	Staging CT	Staging CT
Intermediate (3)	Focused CT thorax	Staging CT
Low (0-2)	Review in clinic	Focused CT Thorax

## Discussion

There is no published data on the use and “hit rate” of pre-clinic MDCT imaging especially in the field of LC. The nearest similar published study was by La Roche et al where the role of CT prior to bronchoscopy was investigated [8]. They concluded that CT prior to bronchoscopy improved diagnostic yield, obliterated the need for further investigations and was cost effective. NICE guidance on LC also supports the performance of staging scan prior to performing bronchoscopy or any other biopsy procedures [10]. If a chest physician assesses the patient prior to MDCT imaging, they invariably are brought back to a second clinic consultation to discuss the results of MDCT and plan for further management. Performing MDCT imaging prior to the clinic review will shorten the diagnostic time line and reduce the number of clinic appointments.

In this setting, the primary question that MDCT needs to confirm the presence or absence of malignancy and/or other significant pathology. In our study, pre-clinic MDCT demonstrated malignancy in 43% of patients while 31% had other significant findings. The MDCT report would facilitate a more informed consultation at the RAL clinic where the physician can discuss the findings, prognosis and arrange appropriate investigations. With this information at hand the physician can discuss the ideal biopsy technique for histological confirmation, assess suitability for radical treatment and arrange counselling or urgent palliative therapy for appropriate patients (bone metastases with impending cord compression or mediastinal disease causing significant collapse). A pre-clinic scan will also suggest the appropriate segmental bronchus for washouts/biopsy with bronchoscopy and improve the diagnostic yield, compared to a bronchoscopy performed ‘blind’ [8]. The same principle would aid clinician’s in performing mediastinotomy and trans-bronchial biopsies.

In our study, 45% of patients did not have lung cancer but had other abnormality. These abnormalities either accounted for the patient’s symptoms or were completely incidental (significant and insignificant). In the former, the clinic visit can be tailored to suit the specific patient by organising correct investigations and starting appropriate treatment, for example, the 16 cases of consolidation in our study. In the latter group, patients were informed of the incidental findings and management decisions were made.

The final group of patients are those who had a normal MDCT prior to the clinic (12%). In these cases, MDCT helped the clinical management as further investigations for clinical symptoms were organised promptly (bronchoscopy for haemoptysis). Our ‘negative’ rate does not compare that well with the study from La Roche who had a negative rate of 4% [8]. The reason for this is not entirely clear and on review, 80% of these cases either had a parenchymal opacity on the CXR or a bulky hilum. Even on retrospect, majority of these patients would have warranted a MDCT even if seen in the clinic prior to the scan. A high ‘hit rate’ (positive MDCT studies) approaching 100% indicates very high threshold for evaluation with MDCT whereby subtle CXR findings will not be evaluated. On the other hand, a lower hit rate would cause unnecessary exposure to radiation. The latter would be of greater concern in younger patients, especially for those under the age of 35 where lung cancer is rare [11].

In our study, CRS significantly predicted the presence or absence of cancer while increasing MCRS was associated with increasing odds of detecting malignancy. Based on this a staging scan is only justified when CRS > 2 and MCRS is high. Patient’s age was not a significant variable in predicting cancer, contrary to common belief and interpolated data from multiple studies on larger pulmonary nodules [12-14]. This may be secondary to a fairly selected sample and/or limited sample size.

To improve our diagnostic yield and guide our future practice we developed an algorithm primarily based on MCRS and the age of the patient. The age criterion was also included in the algorithm to account for lower risk of cancer and minimise radiation exposure in younger patients. This algorithm tries to balance the risk benefit ratio and stratifies patients into two broad groups based on age. We have introduced the concept of a, ‘Focused CT thorax’ this is a low dose MDCT examination without intravenous contrast. The scan only covers the area of abnormality/suspicion on the CXR. A radiologist reviews the images, while the patient is still on the table, and decides if further imaging or staging MDCT is required. This technique has a great potential in imaging patients with a low MCRS or focal abnormality on a CXR. With the use of this algorithm we can improve the sensitivity (78%) and specificity of picking abnormalities on MDCT.

In the normal group, the value of a negative scan is difficult to quantify. Effective radiation dose from an MDCT LC staging ranges from 3 to 8 mSv. These patients also received intravenous contrast thereby exposing them to the potential risk of contrast allergy. We certainly do not support the indiscriminate use of radiation and believe that this practice compares much better than the hit rate of lung cancer screening studies [1, 4-6].

The limitations of this study include the subjective nature of risk assessments by the physicians. However, they all followed common guidelines when assessing the risks [10]. We acknowledge the inherent bias due to lack of randomisation into patients with CT prior to the clinic and the ones without. We presume that performing pre-clinic MDCT imaging will reduce the number of clinic visits and also decrease the diagnostic timeline. This will have significant cost savings and improved compliance with patients. We did not collect specific data to validate this. Our data shows increased incidence of significant abnormalities with increasing age but does not support any clear age cut off in risk stratification. For the algorithm we took 40 years as a cut off as this affords a good balance between risk benefit of radiation and presence of malignancy [15]. The algorithm was tested in the current group of patients but has not been validated prospectively.

In conclusion, MDCT scanning prior to clinic appointment in appropriately triaged patients with suspected LC is feasible and has a promising role in the modern care of lung cancer patients. Pre clinic MDCT also empowers physicians with additional information at the primary consultation. The use of the suggested algorithm will streamline investigation for suspected LC with due consideration of radiation risks and may have significant cost saving.
